# Pan-Genome Analysis Reveals Functional Divergences in Gut-Restricted *Gilliamella* and *Snodgrassella*

**DOI:** 10.3390/bioengineering9100544

**Published:** 2022-10-12

**Authors:** Zhengyi Zhang, Yulong Guo, Fan Yang, Jilian Li

**Affiliations:** Key Laboratory of Pollinating Insect Biology of the Ministry of Agriculture, Institute of Apicultural Research, Chinese Academy of Agricultural Science, Beijing 100093, China

**Keywords:** corbiculate bees, pedigree diversity, evolution, pan-genomics

## Abstract

*Gilliamella* and *Snodgrassella*, members of core gut microbiota in corbiculate bees, have high species diversity and adaptability to a wide range of hosts. In this study, we performed species taxonomy and phylogenetic analysis for *Gilliamella* and *Snodgrassella* strains that we isolated in our laboratory, in combination with published whole-genome. Functional effects of accessory and unique genes were investigated by KEGG category and pathway annotation in pan-genome analysis. Consequently, in *Gilliamella*, we inferred the importance of carbohydrate metabolism, amino acid metabolism, membrane transport, energy metabolism, and metabolism of cofactors and vitamins in accessory or unique genes. The pathway mentioned above, plus infectious disease, lipid metabolism, nucleotide metabolism as well as replication and repair exert a pivotal role in accessory or unique genes of *Snodgrassella*. Further analysis revealed the existence of functional differentiation of accessory and unique genes among *Apis*-derived genomes and *Bombus*-derived genomes. We also identified eight and four biosynthetic gene clusters in all *Gilliamella* and *Snodgrassella* genomes, respectively. Our study provides a good insight to better understand how host heterogeneity influences the bacterial speciation and affects the versatility of the genome of the gut bacteria.

## 1. Introduction

Corbiculate bees, including honeybees, bumblebees, and stringless bees, are important pollinators [1]. The pollination of these species could ensure the stability of local ecosystems and the income of bee farmers through increasing plant diversity and crop yields [2]. However, because of the destruction of the environment and the abuse of antibiotics, the number of these insects decreased sharply, and they were on the edge of extinction [3]. The intestinal microflora in the social bees could benefit the health and fitness of the host by facilitating the host to degrade toxic substances, absorb polysaccharides, and stimulate the immune response [4]. The gut of social bees, such as bumblebees and honeybees, harbors simple and host-specific bacterial phylotypes. Hence, these types of insects could serve as ideal models to study evolution of gut microorganisms and interaction between the host and gut microorganisms [5]. *Gilliamella* and *Snodgrassella* are the dominant genus in the gut of bees. *Gilliamella* grows on top of the *Snodgrassella* layer to form a dense biofilm. It is proved that *Gilliamella* could degrade polysaccharides, forming a nutritional network with other symbionts and the host. By contrast, *Snodgrassella* behaves as the oxidant of carboxylic acid and triggers a host-beneficial immune response [6,7].

Knowledge about the taxonomy of *Gilliamella* and *Snodgrassella* is key to the study of the evolution of these two genus. In the beginning, research classified the above genus based on the 16S rRNA gene [8]. Nevertheless, the deficiencies of this method, such as mismatch of primers and chimerism of PCR products, usually led to low classification characterized with low resolution [9]. By contrast, the taxonomy constructed by the multiple genes and even the whole genome sequence could largely improve the accuracy [10]. For example, the GTDB (Genome Taxonomy Database) of reconstructing the life tree based on 120 single-copy marker genes improves the classification level of uncultured bacteria [11]. Genome-wide ANI (Average Nucleotide Identity) analysis of 9000 prokaryote genomes, through the 95% ANI threshold to define the classification of species, answers the existence of continuity of genetic diversity and clear species boundaries between microbial genomes [12]. Recently, researchers published the systematic classification of *Gilliamella* and *Snodgrassella* based on the whole genome sequences [13]. However, the evolutionary position of the bacterial sequence obtained from newly isolated strains remains unknown.

On the other hand, sociality maintains and promotes host-specific intestinal bacteria specialization. For example, the forager honeybees derived from two colonies in the Agricultural University contained different microbiota. Firmicutes (*Lactobacillus*), actinobacteria (*Bifidobacterium*), and Cyanobacteria were dominant in one of the colonies, while γ-proteobacteria (*Orbales*, *Gilliamella*), α-proteobacteria (*Rhizobiales*, *Bartonella*), and Firmicutes (*Lactobacillus*) were dominant in another colony [14]. The additional possible evidence to support this explanation is that the species differentiation of corbiculate bees is consistent with the pedigree of core intestinal bacteria [15]. Host-associated intestinal bacteria will make changes to adapt to different hosts in their own genomic structure and function. Additionally, the coexistence with other bacteria could also promote the differentiation of functionality niche in the intestinal environment of the same host [16,17]. For example, *Gilliamella* shows diversity in the metabolism of polysaccharides and the ability to utilize sugar substrates [18]. Recent studies have found that *Gilliamella* lives in different intestinal locations and utilizes different nitrogenous waste capabilities of the host [19]. However, we still do not know enough about the functional diversity in genome evolution.

The pan-genome, which was first proposed by Tettlin, is the whole genomic repertoire of a specific species [20]. Specifically, it consists of a core genome, a dispensable genome, and strain-specific genes, where the core genome includes genes that are shared by all members in a given species and the dispensable genome contains accessory genes absent in some strains [21,22]. Generally, core genes are responsible for essential traits of a species. By contrast, accessory genes and unique genes are thought to contribute to species functional diversity based on their influence on species survival in different environment niches (e.g., distinct bacterial host) [23]. At present, pan-genome analysis is the key step to exploring species evolution as well as adaptation given the continuous accumulation of high-throughput genomic sequences. In our study, combined with published *Gilliamella* and *Snodgrassella* genomes, we explored the evolutionary position of six *Gilliamella* strains and five *Snodgrassella* strains that we isolated in our lab and investigated the genomic functional diversity via pan-genomic analyses, carbohydrate enzyme genes annotation, and biosynthesis gene clusters (BGC) analyses. Firstly, it was found that there were 27 different potential species branches for *Gilliamella* and 7 different clades species for *Snodgrassella*, based on ANI and GTDB classification. Secondly, at the genus level, we illustrated the functional diversity of the core genes, accessory genes, and unique genes of the genomes of *Gilliamella* and *Snodgrassella*. Additionally, for both of the above two genus, we revealed the functional differentiation underlying the accessory genes and unique genes between *Apis*-derived genomes and *Bombus*-derived genomes. Finally, we showed the differences in the distribution of biosynthesis gene clusters in genomes of *Gilliamella* and *Snodgrassella*. Taken together, our study showed how host heterogeneity influences the bacterial speciation and affects the versatility of the genome of the gut bacteria.

## 2. Materials and Methods

### 2.1. Bee Sample Collection, Culture, and Identification of Gut Bacteria

A total of seven bees, including one queen, two workers, and one drone of *Bombus terrestris*, as well as one worker of *Bombus lantschouensis*, one worker of *Apis mellifera*, and one worker of *Apis cerana* were collected from different colonies in the Institute of Apicultural Research, Chinese Academy of Agricultural Sciences. The guts of the bee samples were extracted with sterile forceps and then homogenized in Ringer’s solution with a sterile grinding rod. The homogenates were serially diluted to 10^4^ in physiological saline (0.7% NaCl) and plated on heart infusion agar HIA (HIA; Difco BD) and tryptic soy agar TSA (TSA; Difco BD). Then, the plates were incubated at 37 °C in a microaerobically sealed container which was supplied with atmospheres containing 79% N2, 6% CO_2_, and 15% O_2_ atmosphere. After 3 days of incubation, visible colonies were picked up. To quickly identify the target strains belonging to *Gilliamella* and *Snodgrassella*, the genus-specific primer sets were designed based on the genomes of corresponding type strains (*Gilliamella apicola* and *Snodgrassella alvi*) to amplify 16S rRNA genes (*Gilliamella*: the forward primer is 5′-GACGGGTGAGTAATGTATGG-3′ and the reverse primer is 5′-AGGTCGCCTCCCTTTGTAT-3′; *Snodgrassella*: the forward primer is 5′-AATACCGCATACGCCCTGAG-3′ and the reverse primer is 5′-TACGGCTACCTTGTTACGAC-3′). Finally, eleven strains, including six *Gilliamella* and five *Snodgrassella*, were isolated from the intestines of seven bees and identified in the NCBI (National Center for Biotechnology Information) database (Table S1).

### 2.2. Isolates DNA Extraction, Library Preparation, and Sequencing

The genome DNA was extracted from 6 isolates of *Gilliamella* and 5 isolates of *Snodgrassella* according to the protocol of Wizard^®^ Genomic DNA Purification Kit [24]. A total amount of 0.2 ug DNA of each sample was used for the preparation of the DNA library. As recommended by the manufacturer, we used the NEB Next^®^ Ultra™ DNA Library Prep Kit for Illumina (NEB, USA) to generate a sequencing library and added an index code to each sample. In short, the genomic DNA sample was crushed to 350 bp by ultrasound. Then, the DNA fragment was polished, A-tailed, and connected with the full-length connector for Illumina sequencing, followed by further PCR amplification. After the PCR products were purified by a AMPure XP system (Beckman Coulter, Beverly, CA, USA), the concentration of DNA was measured by a Qubit 3.0 Flurometer (Invitrogen, Waltham, MA, USA). The size distribution of the library was analyzed by an Agilent 2100 biological analyzer and quantified by real-time PCR. The DNA library was sequenced on the Illumina platform according to the manufacturer’s instructions, and a paired-end reading of 150 bp was produced.

### 2.3. Genomic Data Collection and Bioinformatics Analysis

The raw data successively underwent quality control and was assembled using the spades.py -1 forward paired-end reads -2 reverse paired-end reads command with the -t 40 and –isolate options (SPAdes v3.15.4) [25]. The assembled genomes of high quality were accessed by QUAST (v5.02) using the quast -l “Genome A, Genome B, Genome C” command with the -t 20 option [26] and CheckM (v1.13) using the checkm lineage_wf command with -t 20 and -x fasta options (Table S2) [27]. In addition to the genomes of intestinal bacteria collected in our laboratory (6 *Gilliamella*, 5 *Snodgrassella*), we collected the published genomes (141 *Gilliamella*, 83 *Snodgrassella*) from the NCBI database for subsequent bioinformatics analysis (Tables S3 and S4). The average nucleotide identity was calculated using the fastANI -ql query.list -rl ref.list command (FastANI v2.0.11) [12]. Then, the cluster analysis was carried out using the bactaxR package [28] in the R v4.1.0 (https://www.r-project.org/, accessed on 1 June 2022). The GTDB-TK toolkit was applied to assign taxonomic classifications to all acquired genomes using the gtdbtk classify_wf command with the --extension fa and --cpus 20 options [29].

*Orbus hercynius DSM 22,228* (Accession: GCA_003634275.1) and *Frischella perrara PEB0191* (Accession: GCA_000807275.1) were selected as outgroups for *Gilliamella*, while *Alysiella crassa NCTC10283* (Accession: GCA_900445245.1) and *Kingella denitrificans NCTC10995* (Accession: GCA_900451365.1) were incorporated as outgroups for Snodgrassella. Single-copy orthologue genes were identified and aligned, respectively, by OrthoFinder (v2.5.4) [30] using the orthofinder command with the -t 20 option and MAFFT (v7.505) [31] using the mafft command with the -op 1.53 and -t 2 options. The IQ-TREE (v 1.6.12) was applied to construct a phylogenic tree using the iqtree command with the -m JTT + F + R10, -bb 1000, -alrt 1000, and -nt AUTO options. The BPGA (Bacterial Pan Genome Analysis v 1.3.0) [32] tool was used to perform pan-genome analysis with the default options and parameters according to the user manual (http://sourceforge.net/projects/bpgatool/, accessed on 1 January 2022). From this process, the KEGG (Kyoto Encyclopedia of Genes and Genomes) pathway was identified at the similarity threshold of 0.5.

The structural and functional annotation was processed using the prokka command with default options and parameters (Prokka v1.14.6) [33]. Specific CAZyme genes annotation was conducted using the run_dbcan.py command with default options and parameters (dbCan2 v2.0.11) [34], and we used algorithms of HMMER [35], DIAMOND [36], and Hotpep [37] with default e values for precise CAZyme annotations. Secondary metabolite BGCs (biosynthetic gene clusters) identification was carried out using the antismash command with the --cb-general, --cb-knownclusters, --cb-subclusters, --asf, --pfam2go, --smcog-trees, --cpus 20, --taxon bacteria, and --genefinding-tool prodigal options (antiSMASH v6.0.1) [38]. The antiSMASH software uses some abbreviations internally to refer to the different types of secondary metabolite clusters (Table S5).

## 3. Results

### 3.1. Population Delimitation within Gilliamella Genus and Snodgrassella Genus Based on ANI and GTDB Analysis

**Figure 1:**
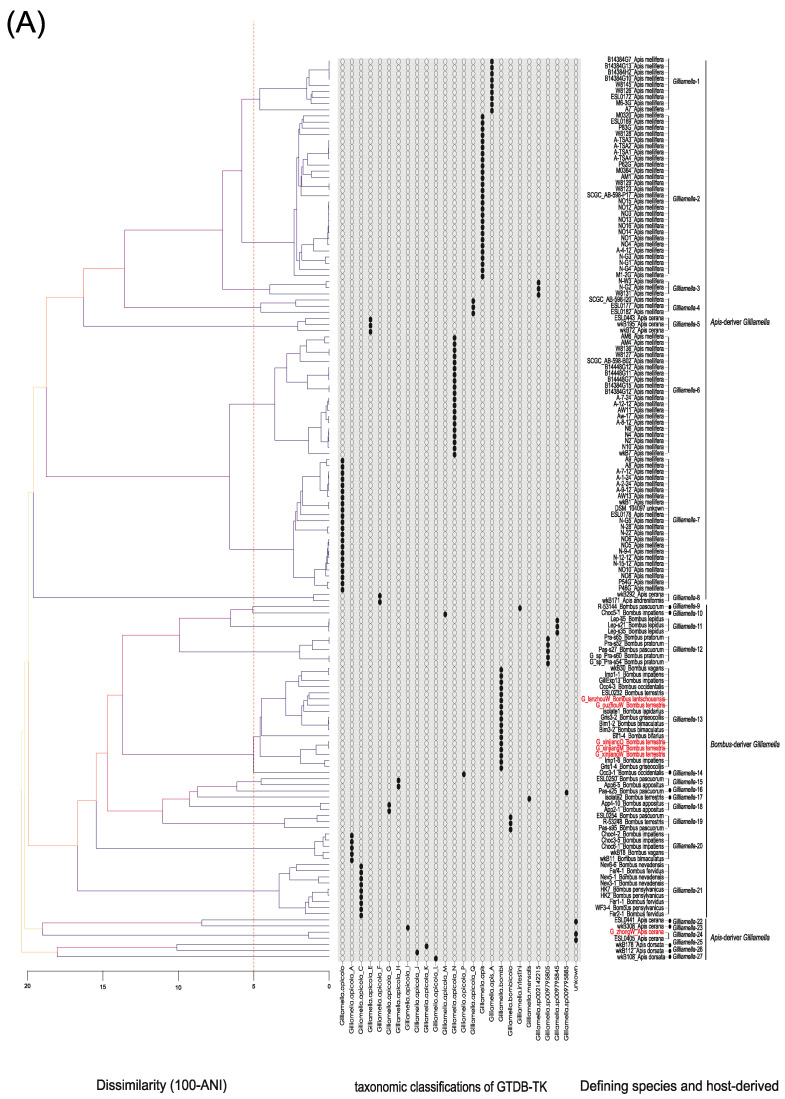

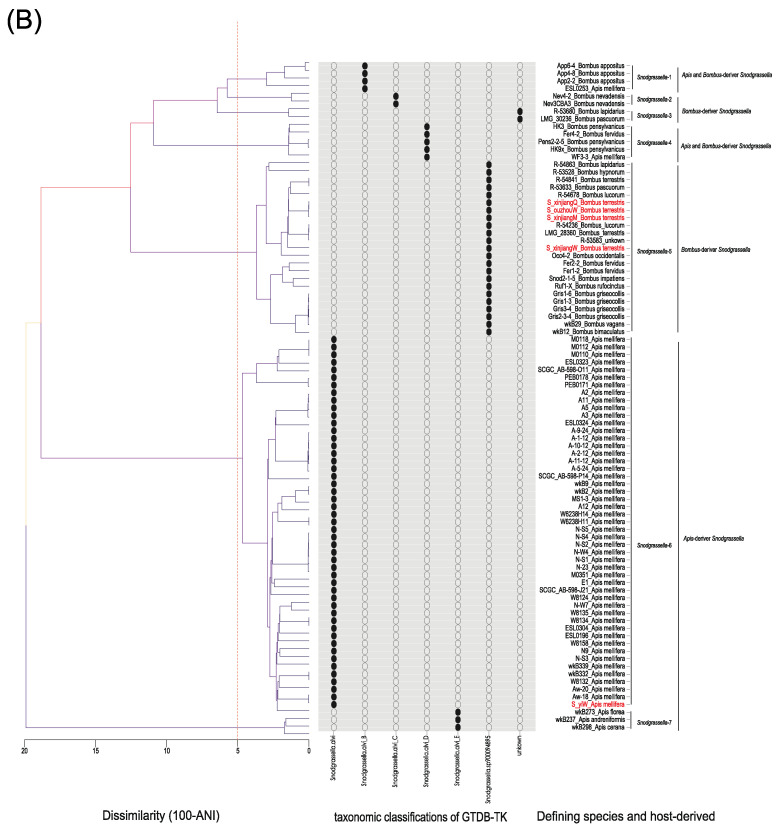
Population delimitation of (**A**) 147 *Gilliamella* strains and (**B**) 88 *Snodgrassella* strains. The right refers to the dendrogram of ANI. The red dashed line displays the 95% ANI for species delineation. The left describes the composition of ANI clades based on a 95% threshold and represents the genus of the host. The middle refers to species classification of GTDB-TK. Red text labels the strains isolated from our lab.

To explore the population diversity within genus *Gilliamella* and *Snodgrassella*, we calculated the genomic similarity based on ANI score and used the GTDB-Tk software toolkit to assign taxonomic classifications for 147 *Gilliamella* isolates and 88 *Snodgrassella* isolates, respectively. Apart from three unclassified genomes of *Gilliamella* isolates, the GTDB database defined the remaining genomes as 25 species. At the intra-species ANI cutoff (>95%), *Gilliamella* genomes were assigned to 27 clades (*Gilliamella* 1–27) whose compositions were identical to the taxonomic classifications of GTDB, except for *Gilliamella* 22 and 24. According to the NCBI database, P62G, wkB1, R-53144, R-53248, bombi isolate 1, and mensalis isolate 2 were the six type strains. These type strains belonged to distinct ANI clades, of which, strains P62G, wkB1, and bombi isolate 1 were included in the top-populated clades *Gilliamella* 2, *Gilliamella* 7, and *Gilliamella* 13, respectively. Strain R-53248 belonged to *Gilliamella* 19 and strain R-53144 and mensalis isolate 2 were involved in the one-branch-based clades *Gilliamella* 9 and *Gilliamella* 17. In addition to type strains, our laboratory strains G_xinjiangQ and G_xinjiangM which belonged to queen and drone, respectively, of *Bombus terrestris*, as well as G_xinjiangW and G_ouzhouW which belonged to the worker of *Bombus terrestris*, were involved in *Gilliamella* 13. Strain G_lanzhouW which isolated from *Bombus lantschouensis* was contained in *Gilliamella* 13 as well. Strain G_zhongW which isolated from *Apis cerana* was contained in *Gilliamella* 24. These results suggest that strain G_zhongW might represent a new species different from the above type strains (Figure 1A).

For *Snodgrassella*, seven clades (*Snodgrassella* 1–7) were clustered based on both ANI score and GTDB database. This number was consistent with the previous classification, although the dataset of our study and the previous study were not totally identical. The type strain wkB2 and our isolates, S_yiW from *Apis mellifera*, were contained in the most populated clade, *Snodgrassella* 6. In addition, our isolated strains, S_xinjiangQ, S_xinjiangM, S_xinjiangW, and S_ouzhouW, which derived from *Bombus terrestris*, were contained in the second most populated clade, *Snodgrassella* 5, which hints that these strains might belong to another new species different from wkB2 (Figure 1B).

Furthermore, for *Gilliamella*, the host of the strains within one clade was totally derived from the same genus but not exactly from the same species, especially for bumblebee-derived strains. Specifically, almost all clades derived from honeybees contained strains of the same host species; however, six clades (*Gilliamella* 12, 13, 15, 19, 20, and 21) derived from bumblebees contained strains belonging to diverse host species. For *Snodgrassella*, most of the strains classified as one clade belonged to the host of the same genus. However, the host of strain from *Snodgrassella* 1 and *Snodgrassella* 4 belonged to honeybees or bumblebees. Except for *Snodgrassella* 2, the strains of the remaining clades that belonged to the same host genus derived from more than one host species. For example, *Snodgrassella* 5 contained 23 strains that were from hosts *Bombus lapidaries*, *Bombus hypnorum*, *Bombus terrestris*, *Bombus pascuorum*, *Bombus lucorum*, *Bombus occidentalis*, *Bombus fervidus*, *Bombus impatiens*, *Bombus rufocinctus*, *Bombus griseocollis*, *Bombus vagans*, and *Bombus bimaculatus*(Figure 1A,B).

### 3.2. Phylogeny Reconstruction for Gilliamella Genus and Snodgrassella Genus

**Figure 2:**
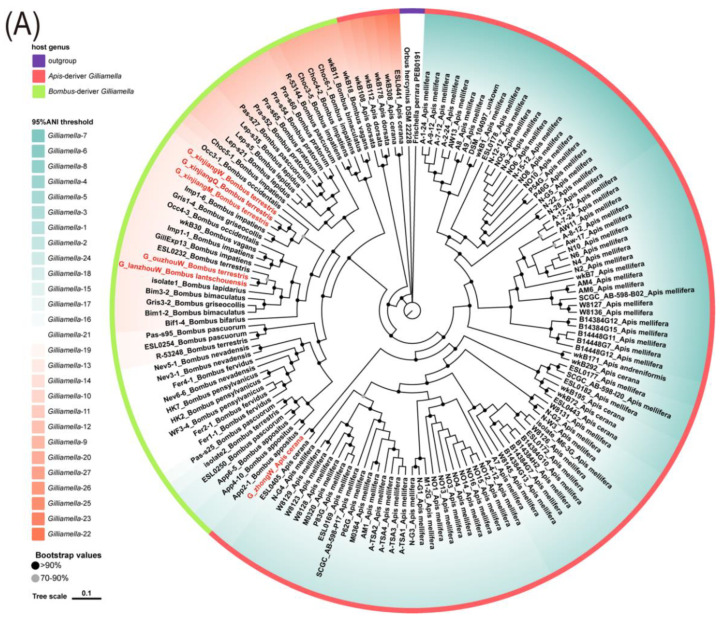

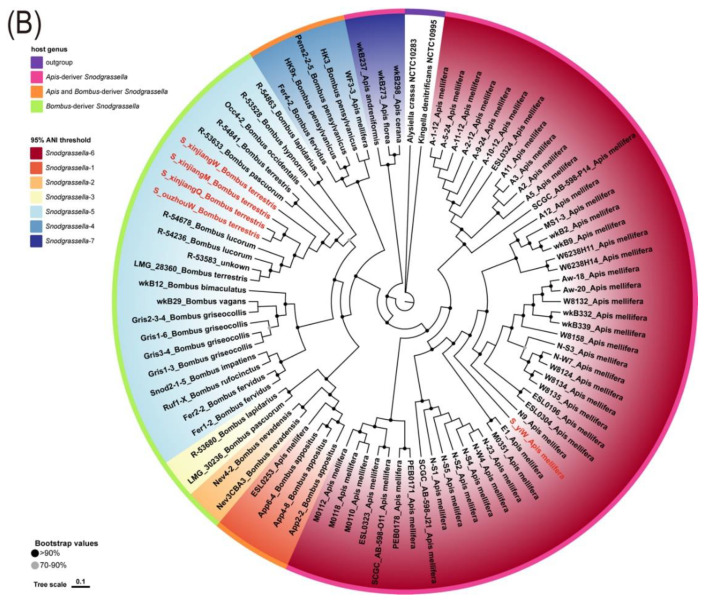
Phologenomic distribution of (**A**) 147 *Gilliamella* strains and (**B**) 88 *Snodgrassella* strains. The bootstrap is presented by the bar, 0.1 divergence. The values are denoted by the color of internal nodes. Each clade is colored according to ANI classification. Red text labels the strains isolated in our lab. The color of the outer ring describes the genus of the host.

The phylogeny tree of *Gilliamella* and *Snodgrassella* were constructed based on single-copy orthologous genes. For *Gilliamella*, strains from the same host genus were more strictly clustered, which is consistent with the results of previous studies. Our isolates G_xinjiangQ, G_xinjiangM, and G_xinjiangW clustered together. In addition, these three strains were closely related to *Bombus occidentalis*-derived strain Occ 3-1 and *Bombus impatiens*-derived strain Imp 1-6. Isolate G_ouzhouW was grouped with G_lanzhouW. The above two strains were adjacent to ESL0232 sampled from *Bombus_terrestris* and isolate1 sampled from *Bombus lapidaries*, respectively. Isolates G_zhongW and ESL0405 which belonged to host *Apis cerana* stemmed from the same branch point (Figure 2A). For *Snodgrassella*, the majority of bumblebee-derived strains and honeybee-derived strains tended to separate from each other. Our isolates S_xinjiangW, S_xinjiangM, S_xinjiangQ, and S_ouzhouW were grouped, of which, S_xinjiangW and S_ouzhouW were neighboring *Bombus pascuorum*-derived R-53633 and *Bombus lucorum*-derived R-54678, respectively. Isolate S_yiW was flanked by *Apis mellifera*-derived N9 and E1. Overall, the phylogenic relationship of our laboratory strain samples reflected the host phylogeny in the genus level but not in the species level (Figure 2B).

### 3.3. Pan-Genome Analysis of Gilliamella and Snodgrassella

**Figure 3:**
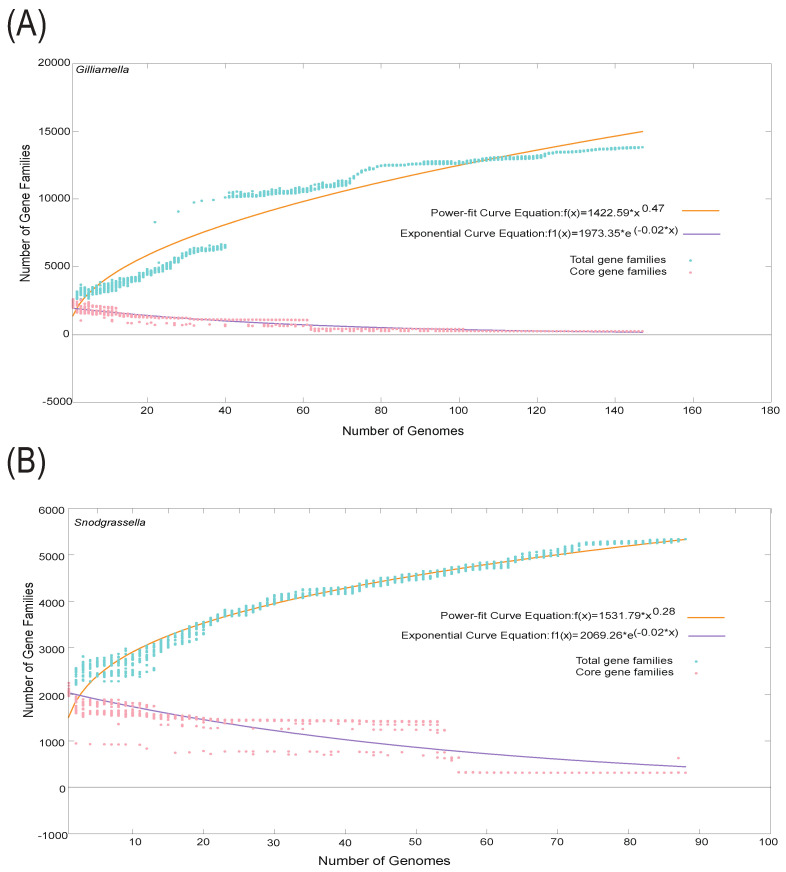

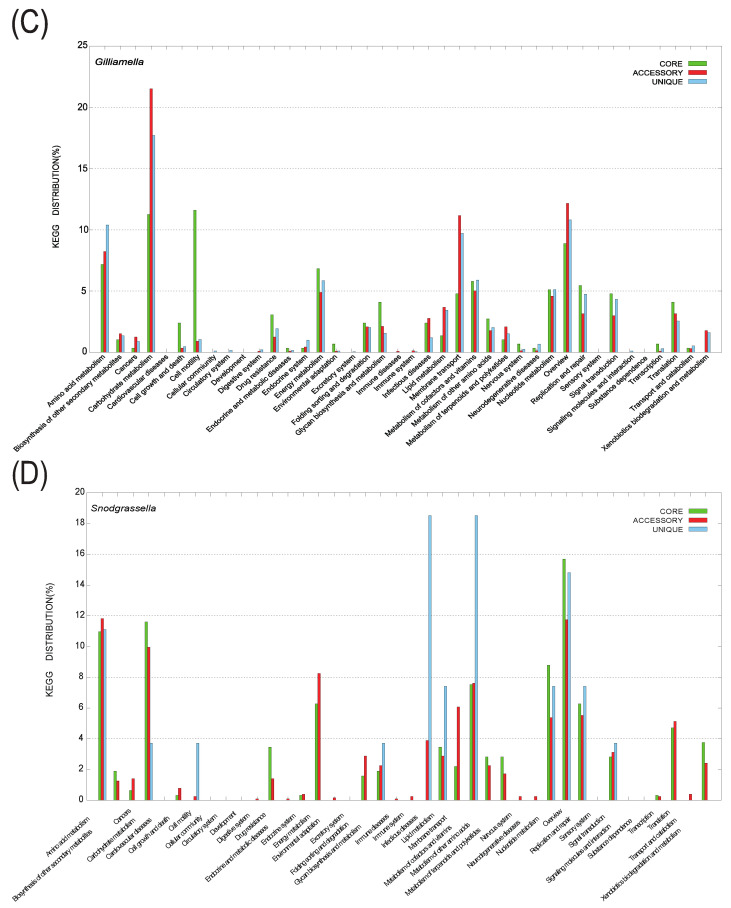
Pan and core genome plot of the analyzed 147 *Gilliamella* genomes (**A**) and 88 *Snodgrassella* genomes (**B**). Graphs shows equations fitting total gene families and core gene families. Distributions of KEGG pathways annotated in core, accessory, and unique genes of 147 *Gilliamella* genomes (**C**) and 88 *Snodgrassella* genomes (**D**).

We conducted pan-genome analysis at different levels for 147 *Gilliamella* genomes and 88 *Snodgrassella* genomes using BPGA software. At the genus level, the pan-genome of *Gilliamella* contained 250 core genes, 7503 accessory genes, and 6071 unique genes (Table S6). In addition, the pan-genome of *Snodgrassella* contained 314 core genes, 4262 accessory genes, and 764 unique genes (Table S7). According to the equations of the core–pan-genome curve, we speculated that the pan-genome of *Gilliamella* is still open and that of *Snodgrassella* is open but may close in the near future (Figure 3A,B).

Functional annotation analysis revealed that the core genes of all the strains of *Gilliamella* were mainly presented in carbohydrate metabolism, cell motility, amino acid metabolism, energy metabolism, metabolism of cofactors, and vitamins. The first five annotated pathways for accessory genes and unique genes were carbohydrate metabolism, amino acid metabolism, membrane transport, energy metabolism, and metabolism of cofactors and vitamins. Genes in pathways of carbohydrate metabolism and membrane transport were more abundant as accessory genes and unique genes than as core genes, while cell motility contained a much larger number of core genes than the other two gene types. These results suggest that, although cell motility plays a vital role for general isolates to survive, functional divergence might not have occurred in this aspect, as *Gilliamella* diversified into distinct lineages. In contrast, the ability of some basic biological processes, especially carbohydrate metabolism and membrane transport, might differentiate across *Gilliamella* through a generate series of accessory genes and unique genes (Figure 3C). For *Snodgrassella*, the function of core genes of the strains was mainly annotated in carbohydrate metabolism, amino acid metabolism, nucleotide metabolism, metabolism of cofactors and vitamins, replication and repair, and energy metabolism. The first five annotated pathways for accessory genes were carbohydrate metabolism, amino acid metabolism, energy metabolism, metabolism of cofactors and vitamins, and membrane transport. The unique genes were apparently involved in infectious disease, metabolism of cofactors and vitamins, amino acid metabolism, lipid metabolism, nucleotide metabolism as well as replication and repair, of which, the ratio for infectious disease and metabolism of cofactors and vitamins were largest among all pathways annotated by three types of genes. These results indicated that, at the genus level, *Snodgrassella* isolates may differ in metabolism of cofactors and vitamins, amino acid, and in the ability to respond to the environment of gut community (Figure 3D).

### 3.4. Pan-Genome Analysis of Gilliamella and Snodgrassella with Different Genus Hosts

**Figure 4:**
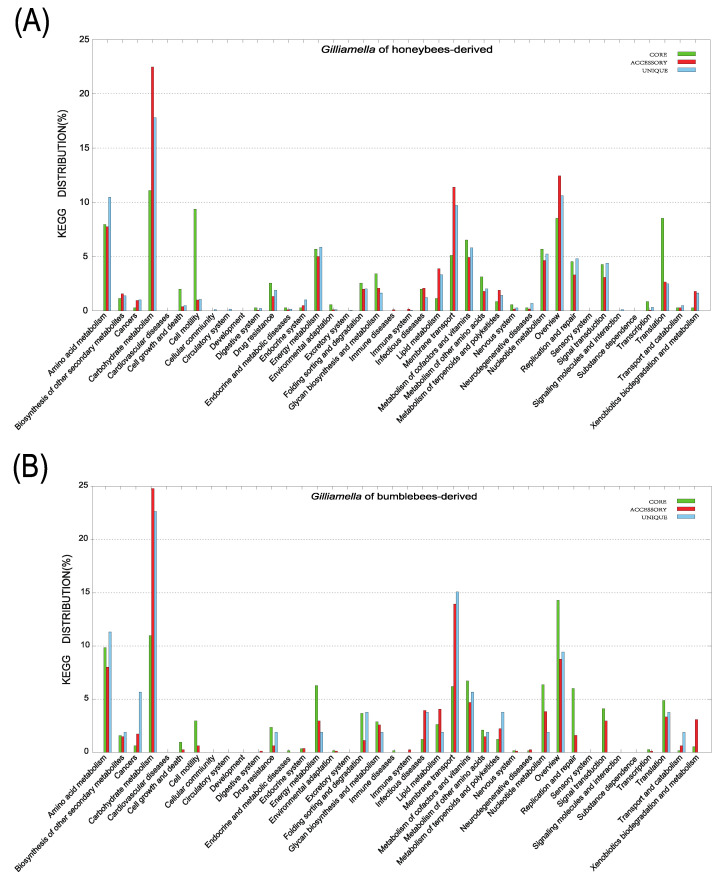

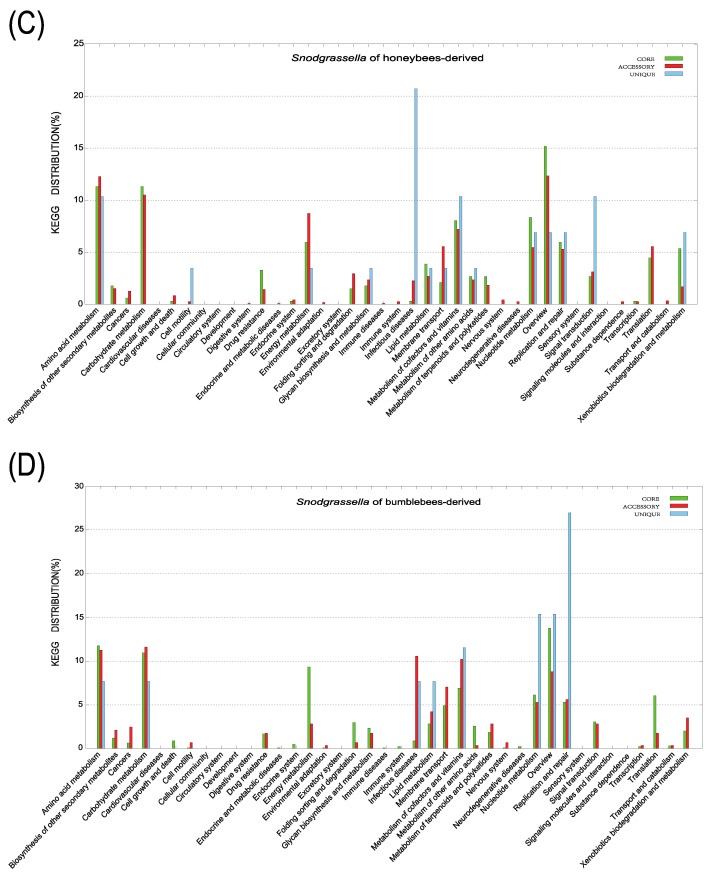
Distributions of KEGG pathways annotated in core, accessory, and unique genes of *Gilliamella* genomes derived from honeybees (**A**) and derived from bumblebees (**B**), as well as *Snodgrassella* genomes derived from honeybees (**C**) and derived from bumblebees (**D**).

Sometimes, the pan-genome size of strains derived from different host can vary because of the differences in the population size and the niche versatility [39]. For *Gilliamella*, the pan-genome of honeybee-derived strains is open, whereas, the pan-genome of bumblebee-derived strains will probably be closed soon (Figure S1A,B). For *Snodgrassella*, both the honeybee-derived strains and bumblebee-derived strains tended to close their pan-genomes (Figure S1C,D). Furthermore, we explored whether the functional differentiation among *Gilliamella* isolates and *Snodgrassella* isolates is associated with host species. Through pan-genome analysis for *Apis*-derived genomes and *Bombus*-derived genomes, we found that for *Gilliamella*, *Apis*-derived isolates possessed more abundant accessory genes or unique genes in energy metabolism and replication and repair, as well as in signal transduction and xenobiotic biodegradation and metabolism, while *Bombus*-derived isolates consisted of more of these two types of genes in membrane transport (Figure 4A,B). For *Snodgrassella*, *Apis*-derived isolates contained many more unique genes in infectious disease and signal transduction, while *Bombus*-derived isolates involved many more unique genes in carbohydrate metabolism and replication and repair, as well as in nucleotide metabolism. These results suggested that functional differentiation among *Gilliamella* isolates and *Snodgrassella* isolates might associate with host species (Figure 4C,D).

### 3.5. Distribution of Genes Related to Carbohydrate Metabolism

**Figure 5:**
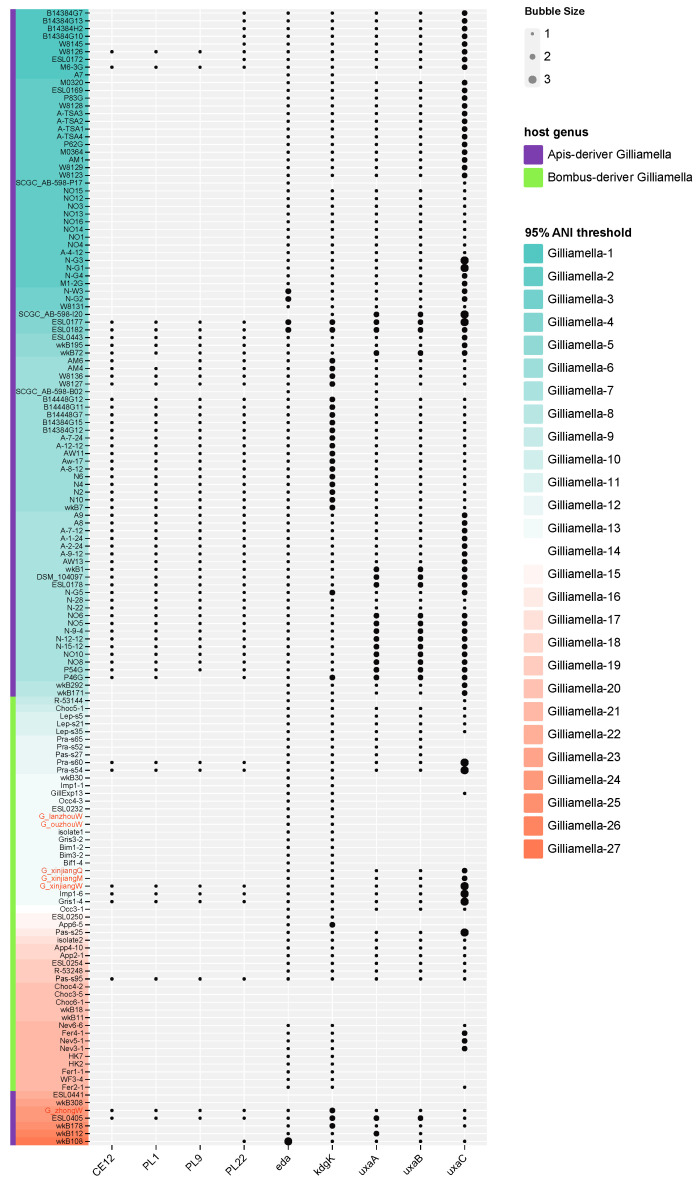
The distribution of key pectin-degrading genes in 147 *Gilliamella* strains. The size of the bubble denotes the gene copy number. The red text marks the strains that were isolated in our lab. The background color of text denotes different ANI clades. The color of the left bar indicates the genus of the host.

We identified the pectin digestion-related genes in the genomes of our isolated *Gilliamella* strains in order to investigate their ability to digest pectin compared with other *Gilliamella* strains. For the pectin lyase gene, isolates G_xinjiangW and G_zhongW possessed the genes CEL12, PL1, PL9, and PL22, while G_xinjiangQ, G_xinjiangM, G_ouzhouW, and G_lanzhouW lost these genes. For galacturonic acid digestion-related genes, isolates G_xinjiangQ, G_xinjiangM, G_xinjiangW, and G_zhongW contained the genes eda, kdgk, uxaA, uxaB, and uxaC, while G_ouzhouW, G_lanzhouW possessed only the eda and kdgk genes. Furthermore, we explored the gene distribution at the species level based on previous ANI analysis. We found gene distribution was different for strains in clades *Gilliamella* 1, 2, 4, 6, 8, 12, 13, 18, and 19, although strains in the each of the above clades were defined as the same species. These results indicated that the ability of pectin degradation could be different among individuals in the same species (Figure 5).

### 3.6. Secondary Metabolite Analysis for Gilliamella Genus and Snodgrassella Genus

**Figure 6:**
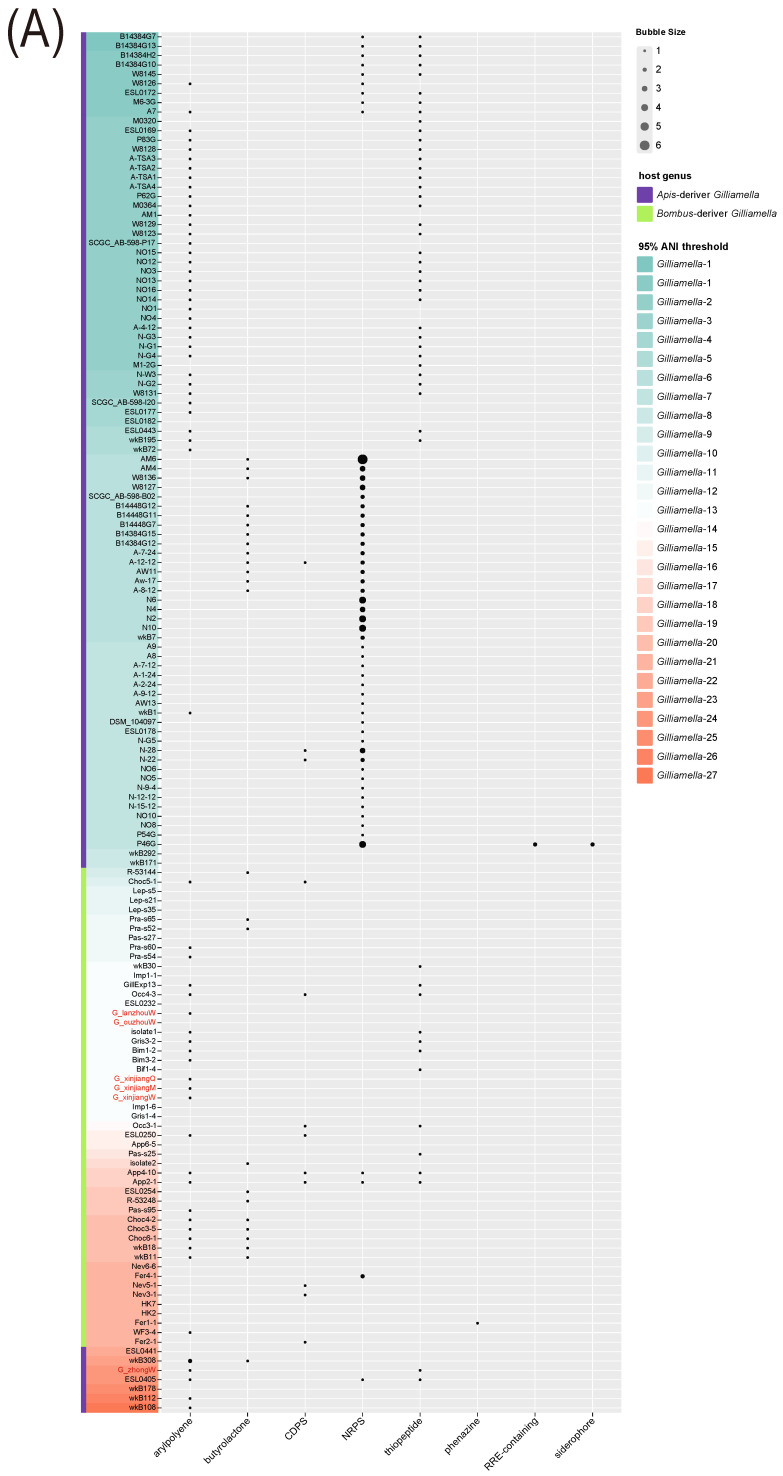

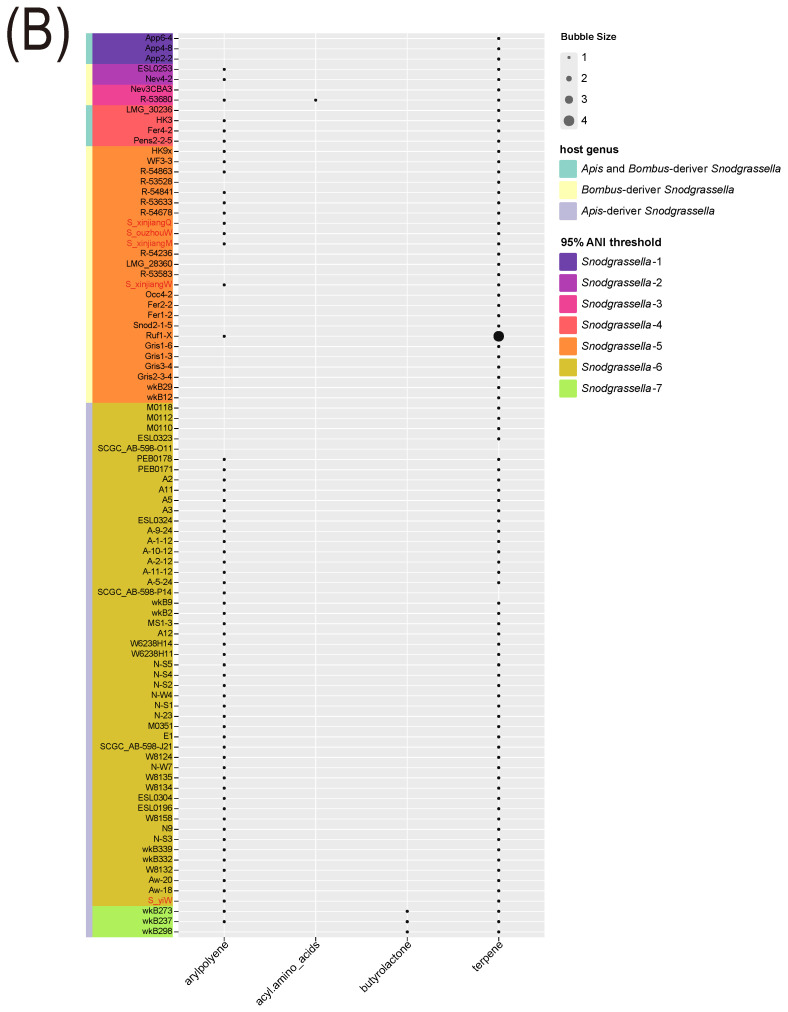
The distribution of biosynthetic gene clusters in the genomes of (**A**) 147 *Gilliamella* strains and (**B**) 88 *Snodgrassella* strains. The red text marks the strains that were isolated in our lab. The background color of text denotes different ANI clades. The color of the left bar indicates the genus of the host.

We explored the distribution of BGCs (Biosynthetic Gene Clusters) in *Gilliamella* and *Snodgrassella*. For *Gilliamella*, eight BGCs were identified in the genomes of 147 strains (arylpolyene, butyrolactone, CDPS, NRPS, thiopeptide, phenazine, RRE-containing, and siderophore). The distribution of the above BGCs was varied among strains of different host species and even among strains defined as the same species in ANI analysis. Arylpolyene, as well as NRPS and thiopeptide, were the most abundant types and they were mainly contained in *Apis*-derived genomes, while CDPS tended to be distributed in *Bombus*-derived genomes. Our isolate G_ouzhouW possessed none of these BGCs; G_xinjiangQ, G_xinjiangM, G_xinjiangW, and G_lanzhouW contained arylpolyene and G_zhongW consisted of both arylpolyene and thiopeptide (Figure 6A).

For *Snodgrassella*, four BGCs were identified in the genomes of 88 strains (arylpolyene, acyl.amino_acids, butyrolactone, and terpene). As *Gilliamella*, the distribution of these BGCs was different among all the strains. Specifically, arylpolyene was frequently located in *Snodgrassella* genomes; terpene was possessed by almost all the strains; butyrolactone was contained only in the genomes of strains belonging to clade *Snodgrassella* 7 which was defined as one independent species in the ANI analysis. Furthermore, we found that all of our isolates, S_ouzhouW, S_xinjiangQ, S_xinjiangM, S_xinjiangW, and S_yiW, contained arylpolyene and terpene (Figure 6B).

## 4. Discussion

Given the development of high-throughput sequencing technologies, analysis of the DNA sequence has become an integral part of the field of bacterial classification. On the basis of the 16S rRNA gene, five species of *Gilliamella* and one species of *Snodgrassella* have been defined (*Gilliamella*: *Gilliamella apicola*, *Gilliamella intestini*, *Gilliamella bombicola*, *Gilliamella bombi*, and *Gilliamella mensalis*; *Snodgrassella alvi*) [8,40]. In our study, alignment of the whole genome sequence (ANI and GTDB) classified *Gilliamella* to 27 species and *Snodgrassella* to 7 species. The number of species we defined was more than the classification of the 16S rRNA gene, indicating a higher degree of speciation of these two dominant genus under long-term nature selection. On the basis of pan-genome analysis, we predicted that the pan-genome of *Gilliamella* is still open. In contrast, the pan-genome of *Snodgrassella* may close in the near future. The openness of the pan-genome might illustrate the prerequisite of genomic adaptation and evolution when coping with a wide range of environments [41]; thus, it usually results in high species diversity. We speculated that the difference in the potential number of species between two, *Gilliamella* and *Snodgrassella*, is probably due to the discrepancy in the degree of openness of their pan-genomes.

This work found that the species’ taxonomic information of our laboratory isolates and that of strains derived from other labs both indicated that strains from different caste and different host species within one genus could belong to the same species; however, strains from the host belonging to different genus tended to be classified as distinct species. These results were consistent with previous findings and might reflect the transmission trend of intestinal bacteria in bees [13,19]. The lineages of *Gilliamella* strains isolated from different castes of the host of the same species in bumblebees were the closest in the results regarding species classification and phylogenetic relationship; the situation in *Snodgrassella* strains isolated from different castes of the host of the same species in bumblebees was the same as that of *Gilliamella*. The above phenomenon was mainly due to the vertical transmission of bumblebees (from queen to unmated queen) and social division of labor, which leads to frequent communication of intestinal bacteria among three types of bumblebees and results in convergence of gut microbiota composition. In fact, there was hardly any gene flow between *Apis* and *Bombus* gut bacteria [16]. Nevertheless, horizontal gene transfer occurred among strains within a host genus [42].

The divergence of species could result from a series of functional differentiation of genomes. In the field of molecular biology, accessory genes and unique genes are considered to have crucial influence on genome evolution and on the interplay between the environment and the genome, being further involved in functional diversification [43,44]. On the basis of the ideas described above, our study illuminated the functional diversity of bacterial strains in *Gilliamella* and *Snodgrassella* based on pan-genome analysis. As core genes, the accessory genes or unique genes of the above genus were significantly involved in “carbohydrate metabolism”, “amino acid metabolism”, “energy metabolism”, and “metabolism of cofactors and vitamins”. Consider “carbohydrate metabolism” in *Gilliamella* as an example: during the long-term co-evolution between host and *Gilliamella*, the efficiency of carbohydrate consumption by different strains correlated with their ability of growth and development. In addition, these strains could degrade the monosaccharides that are difficult for the host to digest and even harmful to the host [6]. In our study, the “carbohydrate metabolism” contained far more accessory genes and unique genes than core genes (Table S8). More importantly, the majority of accessory genes and unique genes in the pan-genomes were aligned to this function (Table S8). These results, on the one hand, were consistent with previous findings that *Gilliamella* is the major bacteria to degrade carbohydrate contained in nectar and pollen, and strains from both the same and different host species differ in their ability to degrade carbohydrates [6,19]. On the other hand, through identifying the variability of the distribution of pectin digestion-related genes among the isolates belonging to the same ANI clades, we emphasized that ability of carbohydrates degradation could be different even among the individuals belonging to the same species of *Gilliamella*. As for synthesis of amino acids, genes related to this function benefit the colonization of *Snodgrassella* by supporting its growth and survival [45]. In our study, the number of accessory genes was much larger than core genes in the category “amino acids metabolism” (Table S9), indicating the function of the degradation and synthesis of amino acids might be due to selection driven by the host. In vivo, *Snodgrassella* utilizes acetate as its energy source, which promotes changes of the methionine biosynthetic pathway [6]. Actually, not only methionine but many other amino acids participate in the production of acetate. For instance, acetate is the major product of histidine deamination. In addition, the breakdown of the basic amino acids arginine and the dissimilatory metabolism of cysteine could also yield acetate [46]. In our data, we noticed the distribution of accessory genes in the metabolism of distinct acetate-forming amino acids. For example, there were 26 and 24 accessory genes in the pathway “Cysteine and methionine metabolism” as well as “Arginine and proline metabolism” (Table S9). For *Snodgrassella*, another source of acetate is TCA cycle in which lipolate and thiamine are two important cofactors required by the key enzyme [47,48]. In our study, there were 10 and 1 accessory genes in “Thiamine metabolism” and “Lipoic acid metabolism”, respectively (Table S9), indicating the selection of utilization of these two cofactors. Based on the above findings, the changes of the gene content related to the metabolism of a series of amino acids and cofactors might be in parallel with the energy niche differentiation process that occurred in the adaptation phase.

For *Gilliamella* and *Snodgrassella*, the accessory genes or unique genes were also clearly annotated in the KEGG sub-category named “membrane transport”, which included the KEGG pathway associated with “ABC transporters”. This pathway contained 131 accessory genes and 113 unique genes derived from *Gilliamella* and 50 accessory genes derived from *Snodgrassella* (Tables S8 and S9). Previous studies revealed that the ABC transporter plays an important role in bacterial biofilm formation [49]. For instance, the quality of biofilm formed by the ABC transporter-deletion mutant strain of *Streptococcus mutans* was apparently decreased compared with the control strain. In bee gut, biofilm synthesis could exert a critical effect on the colonization of the bacteria [45]. Type strain of *Snodgrassella* adhered to the wall of ileum via biofilm [8]. We speculated that biofilm formation might be involved in niche differentiation and adaptation of strains from *Gilliamella* and *Snodgrassella* through changes in genes aligned as ABC transporters.

In addition, in *Snodgrassella*, approximately 18% of unique genes and 50 accessory genes were assigned to “infectious diseases” (Table S9). This category was involved in a series of pathways related to the infection of pathogens. *Snodgrassella* possesses protective ability against pathogens. For example, *S. alvi* could assist the host in challenging *S. marcescens* through activating the expression of antimicrobial peptides [7]. The above protection of *Snodgrassella* against pathogens might be represented as evidence of the existence of specific *Snodgrassella* and host recognition network. In nature, the situation of the susceptibility to infectious disease is different for the host in different areas. For instance, honeybees in the Natural History Museum were infected with *Nosema ceranae*. However, *N. ceranae* and neogregarines were detected in those collected from Marchamalo. Such susceptibility to infectious diseases is, to some extent, associated with host specificity [14]. In our study, accessory or unique genes of *Snodgrassella* were contained in “Pertussis”, “Epithelial cell signaling in Helicobacter pylori infection”, “Legionellosis”, “Salmonella infection”, “Vibrio cholerae infection”, “Vibrio cholerae pathogenic cycle”, “Tuberculosis”, and “Epstein-Barr virus infection” (Table S9). These genes might be possible determinants of host specificity while also representing functional diversity of distinct strains in *Snodgrassella*. In this study, the pan-genome analysis also revealed the differences in the annotation pattern of core genes, accessory genes, or the unique genes across *Apis*-derived genomes and *Bombus*-derived genomes. Consider again “infectious diseases” as an example: two core genes of *Gilliamella* derived from bumblebees were annotated in “Epithelial cell signaling in Helicobacter pylori infection”. However, this term was not included in the pathways annotated by core genes of *Gilliamella* derived from honeybees. Likely, in *Snodgrassella*, the core genes of this bacteria derived from honeybees annotated only in “Epithelial cell signaling in Helicobacter pylori infection”. By contrast, those of *Snodgrassella*-derived bumblebees annotated in many other pathways belonging to the “infectious diseases” category. These results suggest that the *Gilliamella* and *Snodgrassella* derived from the host of different genus might have different representative genes coping with “infectious diseases”. For *Snodgrassella*, *Apis*-derived isolates contained many more unique genes in infectious disease, while *Bombus*-derived isolates involved many more accessory genes. This result hints that the strategies coping with “infectious diseases” might be involved in environmental adaptation of different strains from both honeybees and bumblebees. Taken together, we illustrated a series of functional differentiation in the genomes of *Gilliamella* and *Snodgrassella*.

The BGCs were identified in the genomes *Gilliamella* and *Snodgrassella*. Arylpolyene was abundant in both of the above two genus. Previous work proposed that this molecule could protect bacteria from the damage of reactive oxygen species [50]. It was found that the gut immune and epithelial cells could perform anti-bacterial function through releasing reactive oxygen species to the lumen of the host [51,52]. This reactive oxygen species-mediated killing is an important mechanism in pathogen clearance. However, after long-term selection, some bacteria have evolved to respond to such anti-microbial strategies. For example, *Escherichia coli* could express distinct regulators to defend oxidative stress [53]. In bees, *Gilliamella* and *Snodgrassella* are dominant bacteria. It is possible that the distribution of arylpolyene in the genomes might empower the above genus with the property to tolerate oxidative stress. In *Gilliamella*, butyrolactone, thiopeptide, and NRPS were another three dominant biosynthetic gene clusters, of which, butyrolactone is a signaling molecule in quorum sensing and could mediate host–bacteria interaction [54]. It is proved that butyrolactone suppresses the expression of IL-6 in the murine macrophage cell line (Raw 246.7) and decreases the secretion of IL-8 in the human intestinal epithelial cell line (Caco-2/TC7) [55]. These previous findings confirmed the anti-inflammatory effect of butyrolactone on the host and hint that this metabolite may assist *Gilliamella* in regulating the immune response of the host under inflammatory conditions. Thiopeptide is known as a natural-product antibiotic and has been thought to exert an anti-microbial function in diverse bacteria historically [56]. However, it recently has been proven to stimulate biofilm formation. Specifically, thiopeptide could regulate the expression of biofilm-matrix genes and then lead to an expansion of matrix-producing cells in bacterium *Bacillus subtilis* [57]. This research illustrated that thiopeptide could mediate intraspecies and interspecies bacterial interaction. For gut bacteria in bees, genes related to biofilm formation and extracellular interaction have been identified as a host colonization determinant [45]. Our previous findings make it conceivable that thiopeptide in *Gilliamella* may act as a signal in bacterial communication with other microbes and the host. The NRPS encodes non-ribosomal peptide synthetases, and these gene clusters exhibit diverse biological activities including antimicrobial and iron acquisition [58,59]. In bees, multiple systems for iron uptake also facilitate the colonization of gut bacteria [45]. Therefore, we speculate that NRPS might act as a signaling molecule similar to thiopeptide. In *Snodgrassella*, terpene is another abundant gene cluster. This type of compound has been reported to exhibit multiple biological activities, including antibacterial, antioxidant, and anti-inflammatory [60,61]. Thus, terpene may promote *Snodgrassella* to interact with host and other microbes. Taken together, our study showed how host heterogeneity influences the bacterial speciation and affects the versatility of the genome of the gut bacteria.

## Data Availability

The raw sequence data of our lab-isolated strains have been deposited in the Genome Sequence Archive in BIG Data Center, Beijing Institute of Genomics (BIG), Chinese Academy of Sciences, under accession numbers CRA007760 that are publicly accessible at http://bigd.big.ac.cn/gsa, accessed on 8 August 2022.

## Supplementary Materials

The following supporting information can be downloaded at: https://www.mdpi.com/article/10.3390/bioengineering9100544/s1.
