# Supplementary figures and images for: Pan-Genome Analysis Reveals Functional Divergences in Gut-Restricted Gilliamella and Snodgrassella
Source: Bioengineering (Basel). 2022 Oct 12;9(10):544. doi: 10.3390/bioengineering9100544 (PMC9598484; doi:10.3390/bioengineering9100544)

(A)

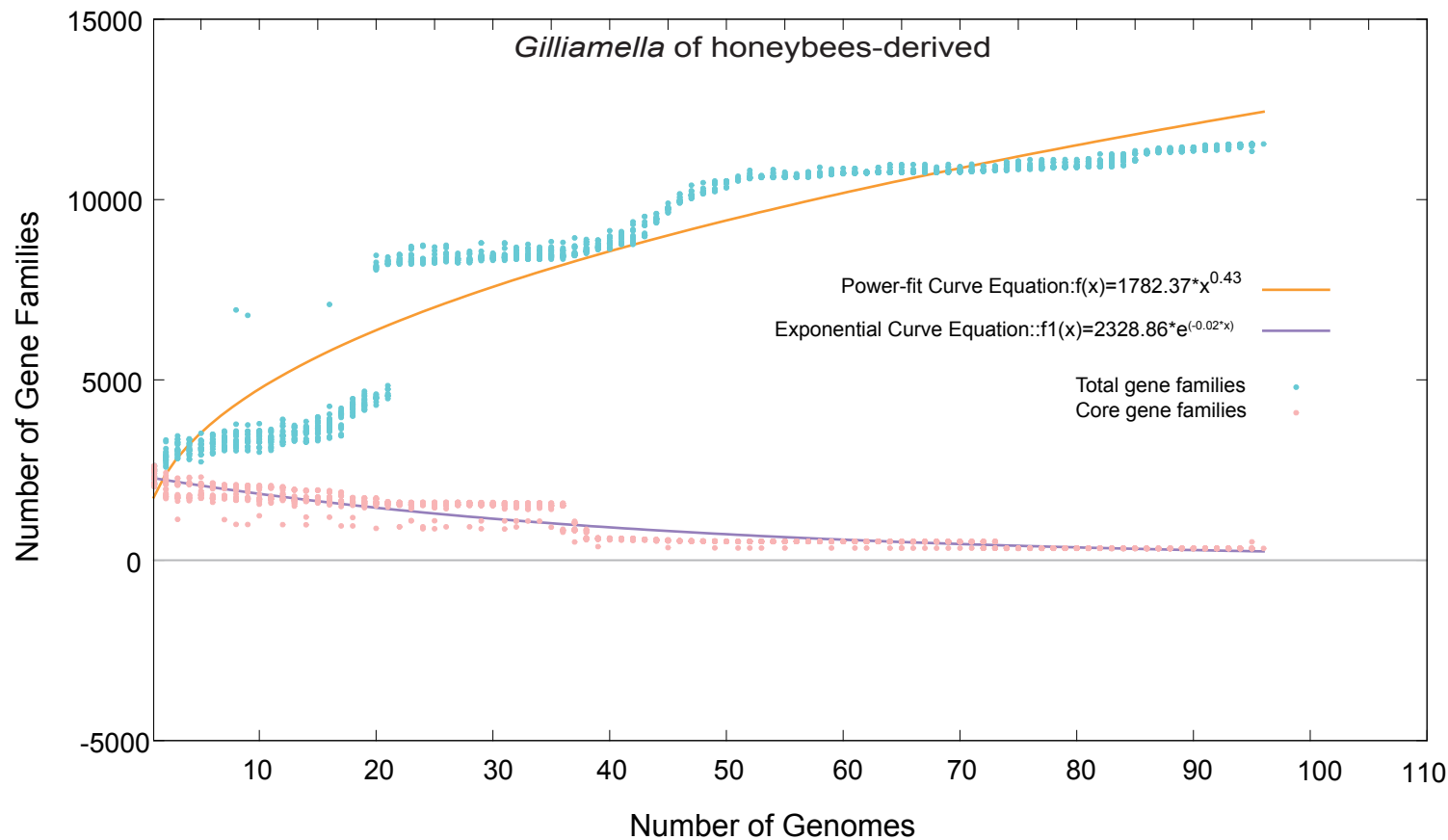

Supplement: Supplementary file 1 [file bioengineering-09-00544-s001.zip › Figure S1A.pdf]

(B)

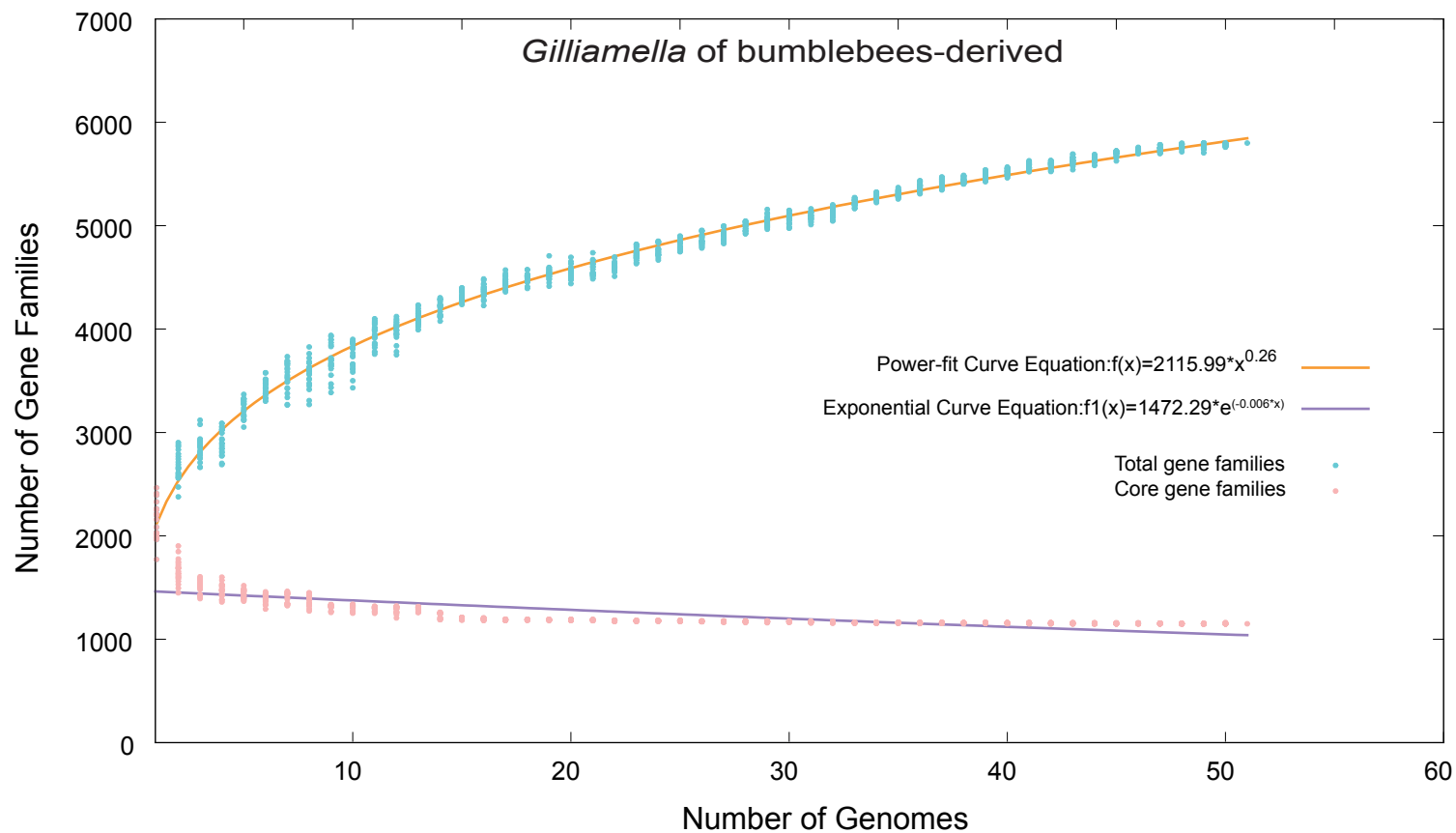

Supplement: Supplementary file 1 [file bioengineering-09-00544-s001.zip › Figure S1B.pdf]

(C)

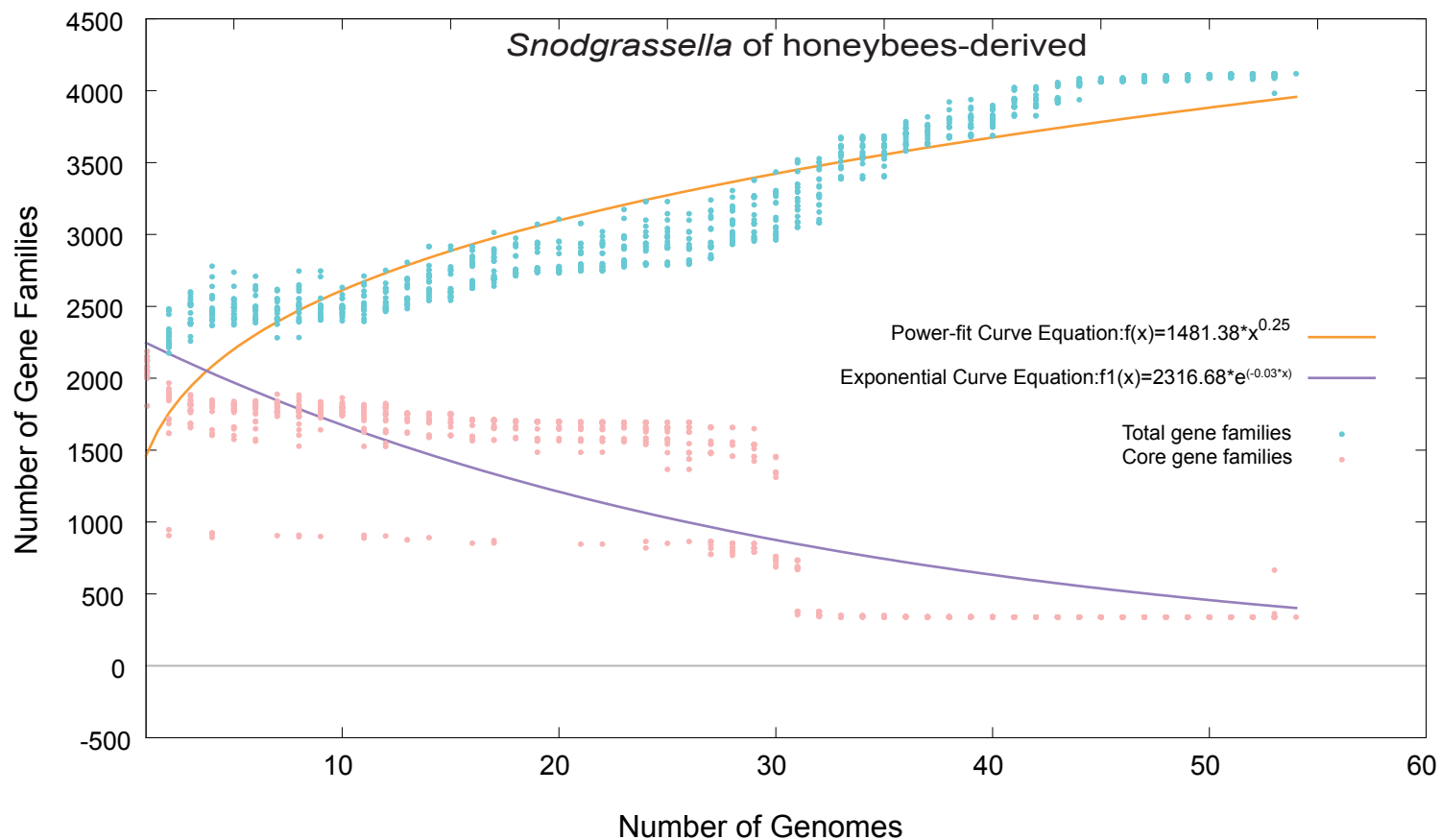

Supplement: Supplementary file 1 [file bioengineering-09-00544-s001.zip › Figure S1C.pdf]

(D)

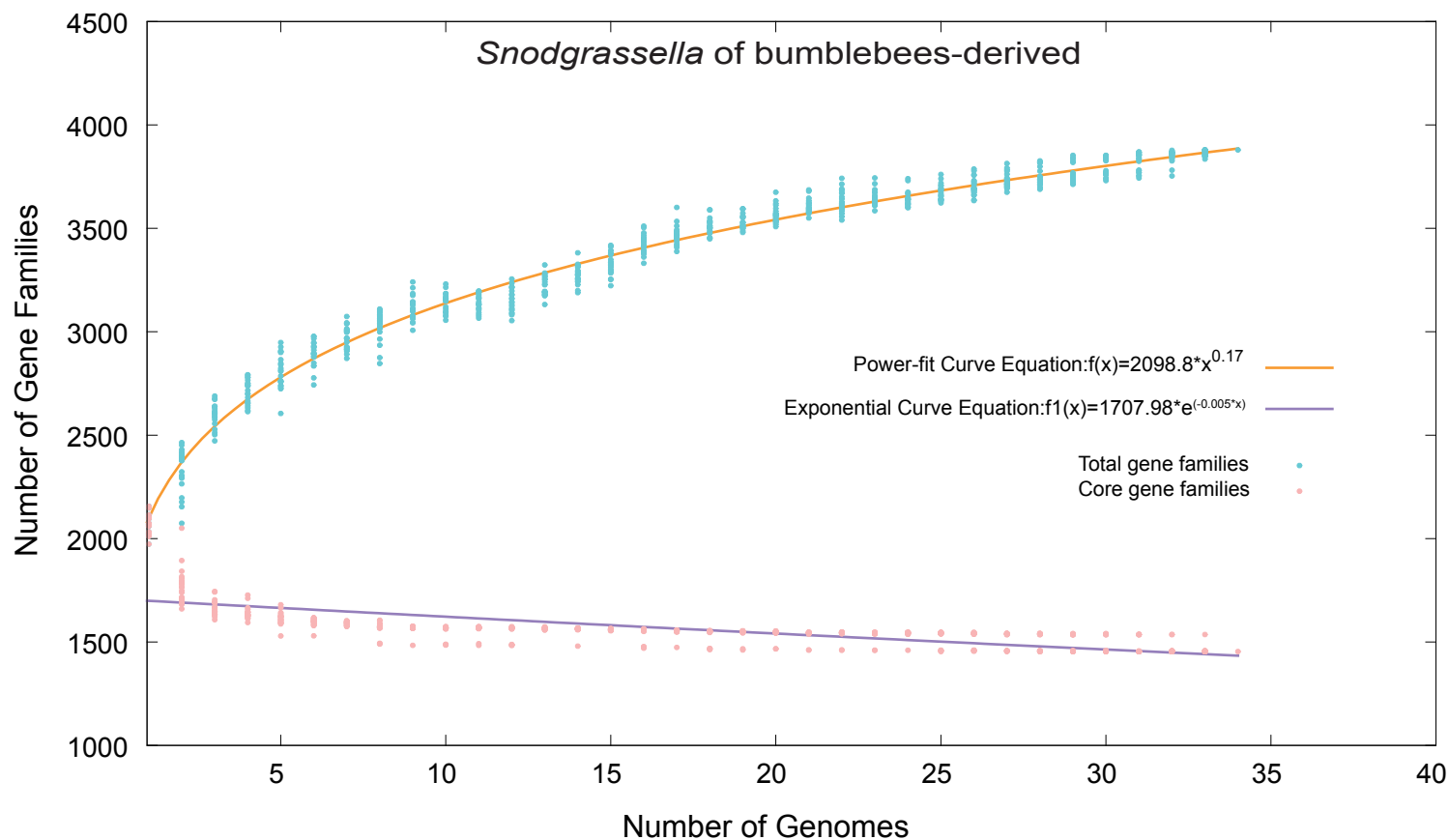

Supplement: Supplementary file 1 [file bioengineering-09-00544-s001.zip › Figure S1D.pdf]
